# Anti-inflammatory potential of *Penicillium brefeldianum* endophytic fungus supported with phytochemical profiling

**DOI:** 10.1186/s12934-023-02091-5

**Published:** 2023-04-27

**Authors:** Asmaa Saleh, Walaa A. Negm, Thanaa A. El-Masry, Duaa Eliwa, Badriyah Alotaibi, Manal E. Alosaimi, Khalid Nijr Alotaibi, Sameh Magdeldin, Sebaey Mahgoub, Engy Elekhnawy

**Affiliations:** 1 Department of Pharmaceutical Sciences, College of Pharmacy, Princess Nourah Bint Abdulrahman University, Riyadh, 84428 Saudi Arabia; 2 Department of Pharmacognosy, Faculty of Pharmacy, Tanta University, Tanta, 31527 Egypt; 3 Department of Pharmacology and Toxicology, Faculty of Pharmacy, Tanta University, Tanta, 31527 Egypt; 4 Department of Basic Health Sciences, College of Medicine, Princess Nourah Bint Abdulrahman University, Riyadh, 84428 Saudi Arabia; 5 Health Services Directorate, Ministry of Defense, Riyadh, 84428 Saudi Arabia; 6 Proteomics and Metabolomics Research Program, Department of Basic Research, Children’s Cancer Hospital 57357, Cairo, 11441 Egypt; 7 Department of Physiology, Faculty of Veterinary Medicine, Suez Canal University, Ismailia, 41522 Egypt; 8 Pharmaceutical Microbiology Department, Faculty of Pharmacy, Tanta University, Tanta, 31527 Egypt

**Keywords:** Carrageenan, Cytokines, LC–MS/MS, Prostaglandin, qRT-PCR, Reactive oxygen species

## Abstract

Various factors contribute to the development of the acute inflammation process, like the pro-inflammatory cytokines, certain enzymes as well as oxidative stress mediators. The anti-inflammatory potential of the endophytic fungus *Penicillium brefeldianum* was explored in carrageenan-induced inflammation in rats. After isolation of the fungus from *Acalypha hispida* leaves, it was identified by 18S rRNA gene sequencing. Then, its phytochemical profile was elucidated using LC–ESI–MS/MS technique. There was a remarkable decrease in the edema weight in the endophytic fungi-treated group (200 mg/kg). Also, this group had few inflammatory cells and thickened epidermis with underlying moderate collagenosis when stained with haematoxylin and eosin. Besides, immunostaining with monoclonal antibodies of cyclooxygenase-2 and tumor necrosis factor alpha showed a decrease in the positive immune cells in the endophytic fungi treated group (200 mg/kg) in relation to the positive control. Interestingly, the levels of the inflammatory as well as oxidative stress markers, including prostaglandin E2, nitric oxide, and malondialdehyde, which are hallmarks of the inflammatory process, considerably diminished (*p* < 0.05) in this group. qRT-PCR was utilised to elucidate the impact of the endophytic fungi treatment on the expression of interleukins (IL-1β and IL-6) genes, which decreased in comparison with the positive control group. Consequently, we can deduce that *P. brefeldianum* endophytic fungus has a promising anti-inflammatory potential and should be extensively studied on a broader range in the near future.

**Graphical Abstract:**

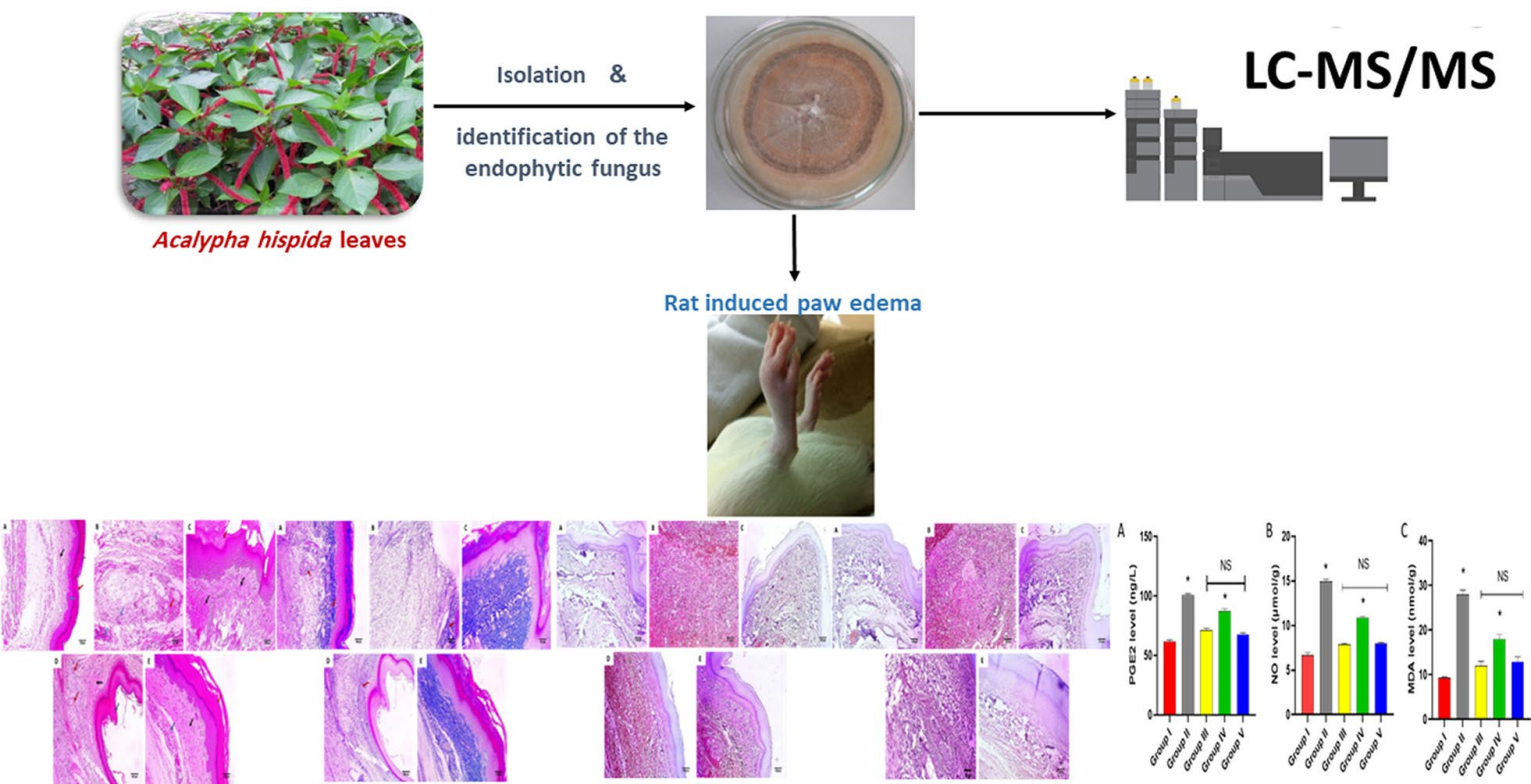

**Supplementary Information:**

The online version contains supplementary material available at 10.1186/s12934-023-02091-5.

## Introduction

Inflammation is a defensive reaction to different persuaders, like infections, wounds, and chemicals. Throughout this progression, various biochemical responses are provoked by some inflammatory mediators [1]. Tumor necrosis factor-alpha (TNF-α), interleukin-6 (IL-6), and interleukin 1 beta (IL-1β) are examples of such pro-inflammatory mediators which start and propagate the inflammatory reactions [2]. In addition, certain enzymes participate in the inflammation reaction, such as cyclooxygenase-2 (COX-2), which have a role in the release of prostaglandins (PGs) [3]. Moreover, reactive oxygen species (ROS) have vital participation in the inflammatory response. Such molecules are released by the cells of inflammation and intensify the inflammatory and oxidative stress reaction [4].

Finding safe and effective anti-inflammatory drugs is challenging owing to the various adverse impacts of the current anti-inflammatory agents, such as non-steroidal anti-inflammatory compounds [5]. The usage of these compounds over long periods, renal, gastrointestinal, and cardiac adverse effects occur. So, we need to reveal novel anti-inflammatory agents.

Recently, natural products have been regarded as a vital source for various pharmaceutical compounds, and their therapeutic effectiveness is being explored in a broad range. This is attributed to their various benefits of efficacy, safety, as well as biocompatibility [6]. Endophytic fungi have a mutually profitable symbiotic relationship with their host plant [7, 8]. It protects against various stresses, such as diseases, insect herbivores, pests, and drought [9, 10] and inhibits the colonization of pathogenic organisms in the host plant [11]. Furthermore, endophytic fungi in the host plant can stimulate growth and improve nutrient recycling [12]. Endophytes are a plentiful source of bioactive chemicals that exhibit interesting pharmacological activities such as antibacterial, antioxidant, anti-diabetic, anti-malarial, and anticancer [13, 14]. The genus *Penicillium* is broadly elucidated owing to its diverse properties [15]. Here, we aimed to find out natural products with promising anti-inflammatory activity from *Penicillium brefeldianum* endophytic fungi.

## Materials and methods

### Chemicals

All chemicals were attained from Merck, USA.

### Collection of plants and isolation of endophytic fungi

The fresh leaves of *Acalypha hispida* (Burm. f.) were gathered from the plantation of Tanta University, Egypt. It was identified by Dr. Esraa Ammar, Plant Ecology Department, Faculty of Science, Tanta University. A voucher specimen (PG-A-END-D-03) was kept at the Pharmacognosy Department at Tanta University. Samples of the plants were rinsed with running tap water and then surfaced sterilized with 70% ethyl alcohol. They were then cut (under sterile conditions) into small parts and imprinted onto agar plates containing potato dextrose agar (PDA) medium supplemented with 250 mg/L amoxicillin. The plates were incubated for 1–2 weeks until adequate growth of the fungus at room temperature. Pure strains of the fungi were attained by repeatedly inoculating the growing fungi on agar plates with new PDA media [16].

### Identification of the endophytic fungi

Pure cultures of *Penicillium brefeldianum* were isolated on PDA plates [17] to be identified by 18S rRNA gene sequencing [18]. The sequence of the utilised primer was 5′-CCTGGTTGATCCTGCCAGTA-3′ in the forward direction and 5′-GCTTGATCCTTCTGCAGGTT-3′ in the reverse order. The sequences of the amplified products were determined at Macrogen Co., Korea. Then, the resulting sequences were put in the Gene Bank (https://blast.ncbi.nlm.nih.gov/Blast.cgi). We used BLAST tool to detect the sequence homology with the closest fungal strains. Using MEGA 7.0 program, a phylogenetic tree was constructed.

### Preparation of the fungal extract

A small part from the fresh mycelia of *Penicillium brefeldianum* was transferred under sterile conditions to a pre-autoclaved cotton-plugged Erlenmeyer flask containing 100 g Asian rice in 110 mL sterile water. It was grown under static conditions at 25 °C for 28 days away from light.

The endophytic fungi were extracted using 99% ethyl acetate by the maceration method. Each extraction was conducted for 24 h, and the process was performed thrice. The filtrates were combined and concentrated to dryness by a rotary vacuum evaporator at 50 °C to obtain a dried extract (10% yield) and prepared for further phytochemical and biological assays [16].

### LC–ESI–MS/MS

LC–ESI–MS/MS analysis of *P. brefeldianum* extract was carried out as previously reported [6, 19]. Both negative and positive electrospray ionization modes were utilized to detect the various phytoconstituents of the *P. brefeldianum* extract. Targeted constituents were determined by comparing LC/MS data with previously published literature and reference databases [20]. PeakView™ software compared retention time and *m/z* values obtained by MS_1_ and MS_2_ [21].

### Anti-inflammatory assay

#### Animals

Fifty male Wistar albino rats weighted 190 to 220 g were used in the current study. The Research Ethical Committee (TP/RE/3/23p-0011) of the Faculty of Pharmacy, Tanta University, Egypt, approved the test.

### Experimental protocol

Inflammation was trigged in the right rat hind paws via subcutaneous (SC) injection of carrageenan solution (0.2 mL). The left hind paws of the rats weren't injected (control) [22]. Animals were randomly grouped into five groups (each comprising ten animals). Normal control (group I) was given 0.9% saline orally. The positive control (group II) was inflamed and given 0.9% saline orally. The standard drug (group III) was inflamed, and celecoxib (50 mg/kg) was given orally. Endophytic fungi treated (groups IV and V) were inflamed and given endophytic fungi (100 and 200 mg/kg, respectively) orally. These two doses were chosen based on the toxicity induced by the higher dose at 300 mg/kg. Then, the animals were anaesthetized and euthanized after four hours. The left and right paws were cut, and their weights were determined. In order to determine the average weight of edema, we determined the difference between the right and left paw weights as previously described [6].

### Histological studies

After preserving the paw tissues in formalin solution to be fixed, they were put in paraffin wax, spliced into thin sections, and stained using hematoxylin and eosin (H&E) [23] and Masson’s trichrome stain [24]. Photos were taken after examination of these sections using a light microscope.

### Immunohistochemical studies

COX-2 and TNF-α immune expression was elucidated by ABclonal Technology kits (Massachusetts, USA) to stain the paw tissues with monoclonal antibodies. Scores, from 0 to 3, were given according to the percentages of the positive staining, as previously reported [25].

#### ELISA

Prostaglandin E2 (PGE2) level was determined in the paw tissues by an ELISA kit (Creative-Biolabs, USA) at 450 nm as designated by the manufacturer.

#### Colorimetric assay

Nitric oxide (NO) as well as malondialdehyde (MDA) levels were detected in the paw tissues by Biodiagnostic colourimetric kits (Egypt) at 540 nm as designated by the producer.

#### qRT-PCR

The gene expression of the mediators of inflammation (IL-1β and IL-6) was detected in the paw tissues using qRT-PCR using the β-actin gene as a housekeeping gene [26]. The primer sequences are revealed in Additional file 1: Table S1 [27].

### Statistics

The achieved assays were conducted in triplicates, and the results are revealed as the mean ± standard deviation (SD) using Graph-Pad Software (prism 8). The significance level was regarded at *p* < 0.05.

## Results

### *P. brefeldianum* endophytic fungus

According to the molecular identification using 18S rRNA of the isolated endophytic fungus (Additional file 1: Fig. S1), it was revealed as *P. brefeldianum.* The results of the DNA sequencing were submitted to GenBank (with an accession number of ON100822) (Table 1).

**Table 1:** Identification of *Penicillium brefeldianum* endophytic fungus using 18S rRNA

Accession number	Identification	Highly similarity isolates	The accession number of highly similar isolates	Identity %
ON100822	*Penicillium brefeldianum* isolate	*Penicillium brefeldianum EBT-1* genes for ITS1, 5.8S rRNA, ITS2, 28S rRNA, partial and complete sequence	LC475454.1	99.09

### Phytochemical investigation

**Fig. 1:**
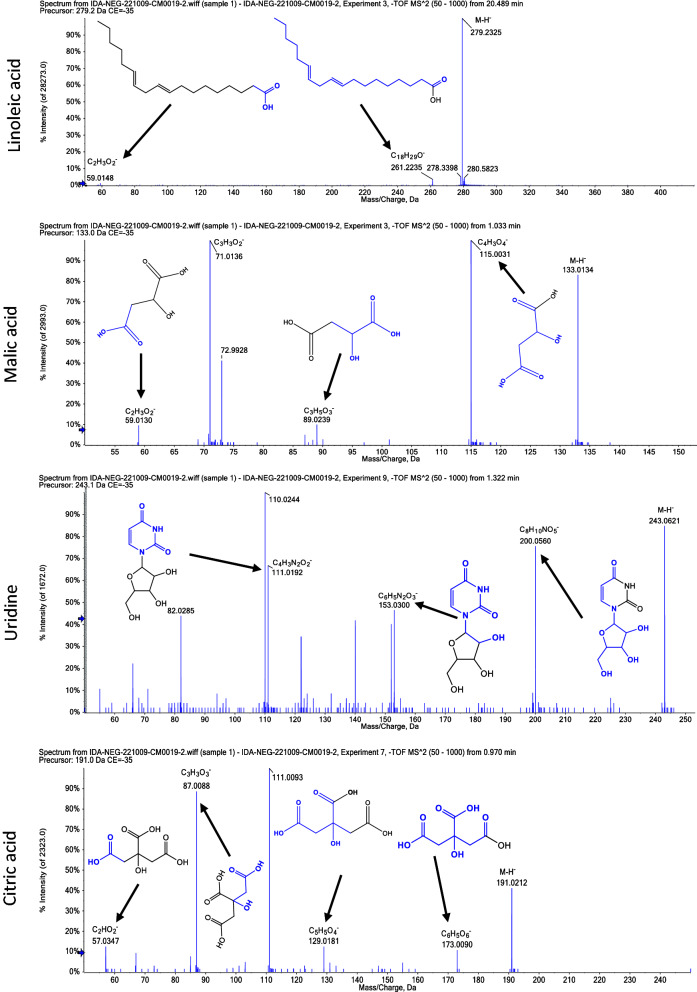
Mass/mass spectra showed a fragmentation pattern of most abundant compounds in negative mode

**Fig. 2:**
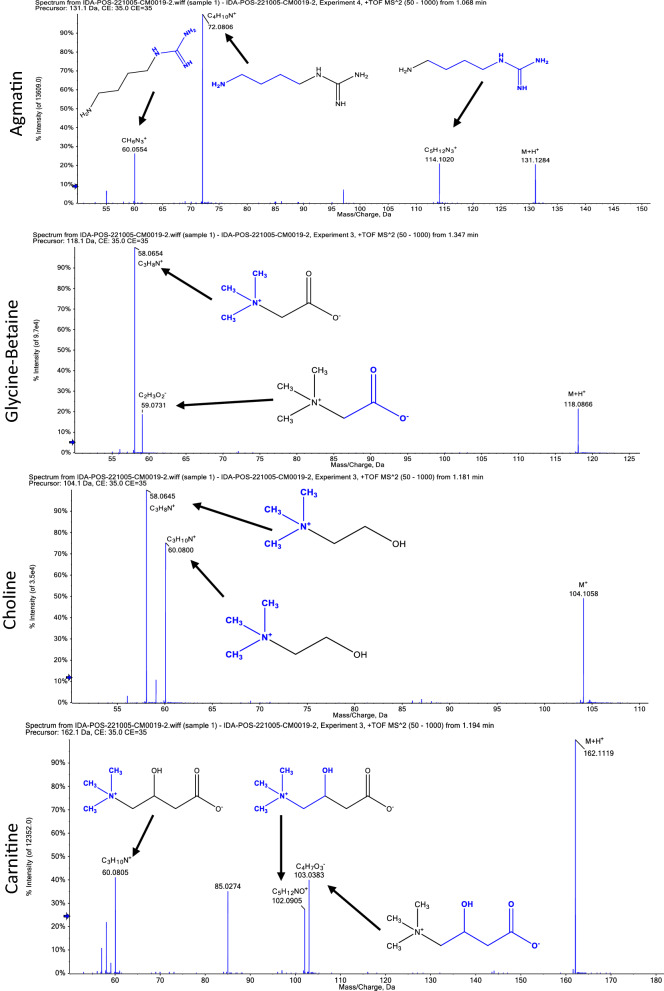
Mass/mass spectra showed a fragmentation pattern of most abundant compounds in positive mode

Twenty-seven compounds were revealed in *Penicillium brefeldianum* extract using LC–ESI–MS/MS in positive and negative modes. The main substances are amino acids, carboxylic acids, and xanthine derivatives. The metabolite profile is presented in Table 2 and Additional file 1: Figs. S2 and S3. While Figs. 1 and 2. showed Mass/Mass spectra displayed the pattern of some selected metabolites’ fragmentation.

**Table 2:** List of tentatively identified metabolites in *P. brefeldianum* extract analyzed by LC–ESI–MS/MS

No	Rt (min)	Precursor m/z	Error ppm	Name	Formula	Adduct ion	MS/MS spectrum	Ontology
1	0.97	191.0218	0.5	Citric acid	C_6_H_8_O_7_	[M−H]^−^	173.0090, 129.0181, 87.0088, 57.0347	Tricarboxylic acids and derivatives
2	1.03	133.0131	0.4	Malic acid	C_4_H_6_O_5_	[M−H]^−^	115.0031, 89.0239, 71.0136, 59.0130	Beta hydroxy acids and derivatives
3	1.07	131.1291	− 0.6	Agmatine	C_5_H_14_N_4_	[M+H]^+^	114.1020, 72.0806, 60.0554	Guanidines
4	1.09	189.1604	0.5	Laminine	C_9_H_20_N_2_O_2_	[M+H]^+^	130.0848, 84.0809, 60.0808	l-Alpha-amino acids
5	1.14	146.0447	0	l-Glutamic acid	C_5_H_9_NO_4_	[M−H]^−^	128.0343, 102.0556	Glutamic acid and derivatives
6	1.16	195.0518	− 0.2	Gluconic acid	C_6_H_12_O_7_	[M−H]^−^	176.9354, 87.0083, 75.0087, 59.0143	Medium-chain hydroxy acids and derivatives
7	1.18	104.1068	− 6.1	Choline	C_5_H_14_NO	[M]^+^	60.0800, 58.0645	Cholines
8	1.25	146.1171	− 0.4	l-beta-Homoisoleucine	C_7_H_15_NO_2_	[M+H]^+^	87.0420, 60.0792, 58.0646	Beta amino acids and derivatives
9	1.29	130.0514	0.8	Leucine	C_6_H_13_NO_2_	[M−H]^−^	84.0765, 61.9892	Leucine and derivatives
10	1.32	243.0613	− 0.7	Uridine	C_9_H_12_N_2_O_6_	[M−H]^−^	200.0560, 153.0300, 111.0192	Pyrimidine nucleosides
11	1.34	163.0612	− 0.5	l-(+)-Rhamnose	C_6_H_12_O_5_	[M−H]^−^	101.0240, 85.0301, 71.0130, 59.0141	Hexoses
12	1.34	341.1043	0.1	Sucrose	C_12_H_22_O_11_	[M−H]^−^	179.0570, 89.0247, 59.0143	O-glycosyl compounds
13	1.35	118.0863	− 0.9	Glycine–betaine	C_5_H_11_NO_2_	[M+H]^+^	59.0731, 58.0654	Alpha amino acids
14	1.38	137.0431	0	Hypoxanthine	C_5_H_4_N_4_O	[M+H]^+^	110.0356, 94.0406	Hypoxanthines
15	1.39	135.0324	− 0.7	Hypoxanthine	C_5_H_4_N_4_O	[M−H]^−^	92.0251, 65.0136	Hypoxanthines
16	1.39	151.0254	0.4	Xylitol	C_5_H_12_O_5_	[M−H]^−^	101.0242, 89.0239, 71.0139, 59.0139	Sugar alcohols
17	1.47	113.0303	− 0.5	Uracil	C_4_H_4_N_2_O_2_	[M+H]^+^	96.0086, 70.0285, 68.0122	Pyrimidones
18	1.62	124.0383	− 0.3	Nicotinic acid	C_6_H_5_NO_2_	[M+H]^+^	106.0291, 80.0482, 78.0331	Pyridine carboxylic acids
19	1.74	130.0495	− 1.1	l-5-Oxoproline	C_5_H_7_NO_3_	[M+H]^+^	84.0437, 56.0493	Alpha amino acids and derivatives
20	1.94	162.1119	0.2	Carnitine	C_7_H_15_NO_3_	[M+H]^+^	103.0383, 102.0905, 60.0805	Carnitines
21	2.08	164.0723	− 0.9	Phenylalanine	C_9_H_11_NO_2_	[M−H]^−^	147.0453, 103.0554, 72.0091	Phenylalanine and derivatives
22	3.63	298.0935	0.6	5′-Methylthioadenosine	C_11_H_15_N_5_O_3_S	[M+H]^+^	136.0611, 119.0346, 61.0104	5′-Deoxy-5′-thionucleosides
23	6.35	211.1442	− 0.9	3-(2-Methylpropyl)-2,3,6,7,8,8a-hexa hydro pyrrolo[1,2-a] pyrazine-1,4-dione	C_11_H_18_N_2_O_2_	[M+H]^+^	183.1483, 114.0921, 98.0587, 70.0650	Alpha amino acids derivatives
24	6.98	208.0959	− 0.8	*N*-Acetyl phenylalanine	C_11_H_13_NO_3_	[M+H]^+^	166.0853, 162.0899, 120.0800, 103.0534	Phenylalanine and derivatives
25	7.02	197.1163	− 0.3	6-Hydroxy-4,4,7a-tri methyl-5,6,7,7a-tetrahydro benzofuran-2(4H)-one	C_11_H_16_O_3_	[M+H]^+^	179.1062, 153.0676, 95.0850, 55.0524	Benzofurans
26	18.05	277.2159	0.1	Linolenic acid	C_18_H_30_O_2_	[M−H]^−^	208.9232, 102.9573, 71.0152	Lineolic acids and derivatives
27	20.49	279.2324	− 0.3	Linoleic acid	C_18_H_32_O_2_	[M−H]^−^	261.2235, 59.0148	Lineolic acids and derivatives

### In vivo anti-inflammatory study

#### The average weight of paw edema

**Fig. 3:**
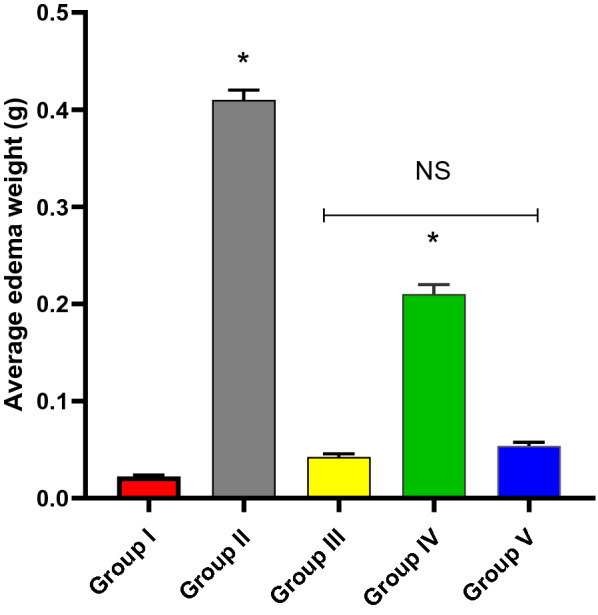
Average paw edema weight of the experimental groups. The symbol (*) reveals a substantial difference (*p* < 0.05) between group V and groups II and IV. NS indicates a non-substantial difference (*p* > 0.05) between groups III and V

The impact of the endophytic fungi on the average weight of paw edema was revealed (Fig. 3). Group V exhibited a substantial decline (*p* < 0.05) in the average paw edema weight compared to groups II and IV.

### Histological assessment

**Fig. 4:**
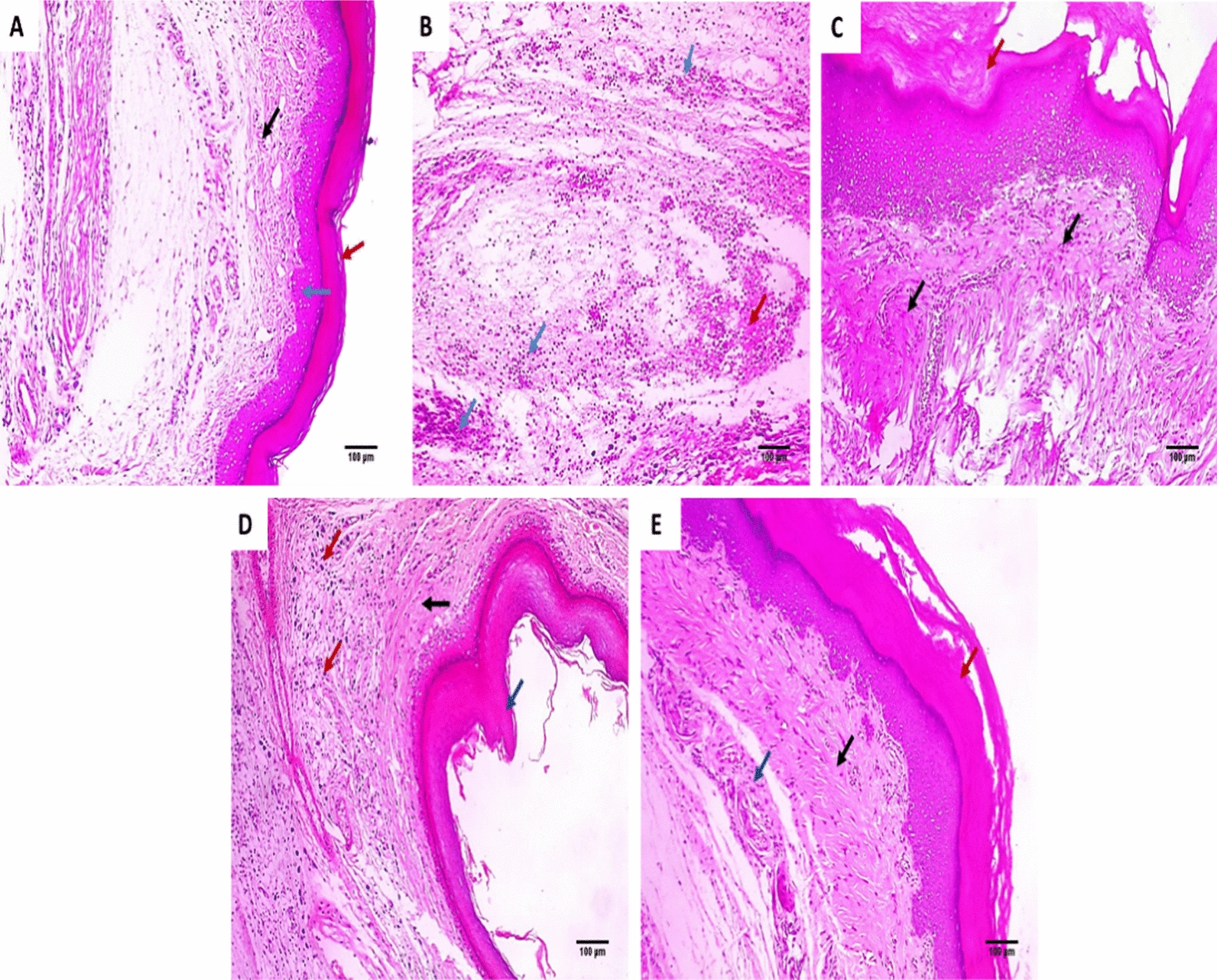
Paw sections stained with H&E: **A** normal control group revealing normal skin consisting of the epidermis of the average thickness (blue arrow) lined with thick keratin (red arrow) and underlying normal dermis (black arrow) (×100). **B** Positive control group revealing skin ulceration filled with a mixture of acute and chronic inflammatory cells (blue arrows) mixed with necrotic debris (red arrow) (×100). **C** Standard drug group revealed no inflammation, and the epidermis was thickened and covered with excessive keratosis (red arrow) with underlying marked collagenosis (black arrows) (×100). **D** Endophytic fungi group (100 mg/kg) revealing moderate dermal inflammation (red arrows), the epidermis was thickened and covered with mild keratosis (blue arrow) with underlying mild collagenosis (black arrow) (×100). **E** Endophytic fungi group (200 mg/kg) revealed few inflammatory cells (blue arrow), and the epidermis was thickened with keratosis (red arrow) as well as underlying moderate collagenosis (black arrow) (×100)

**Fig. 5:**
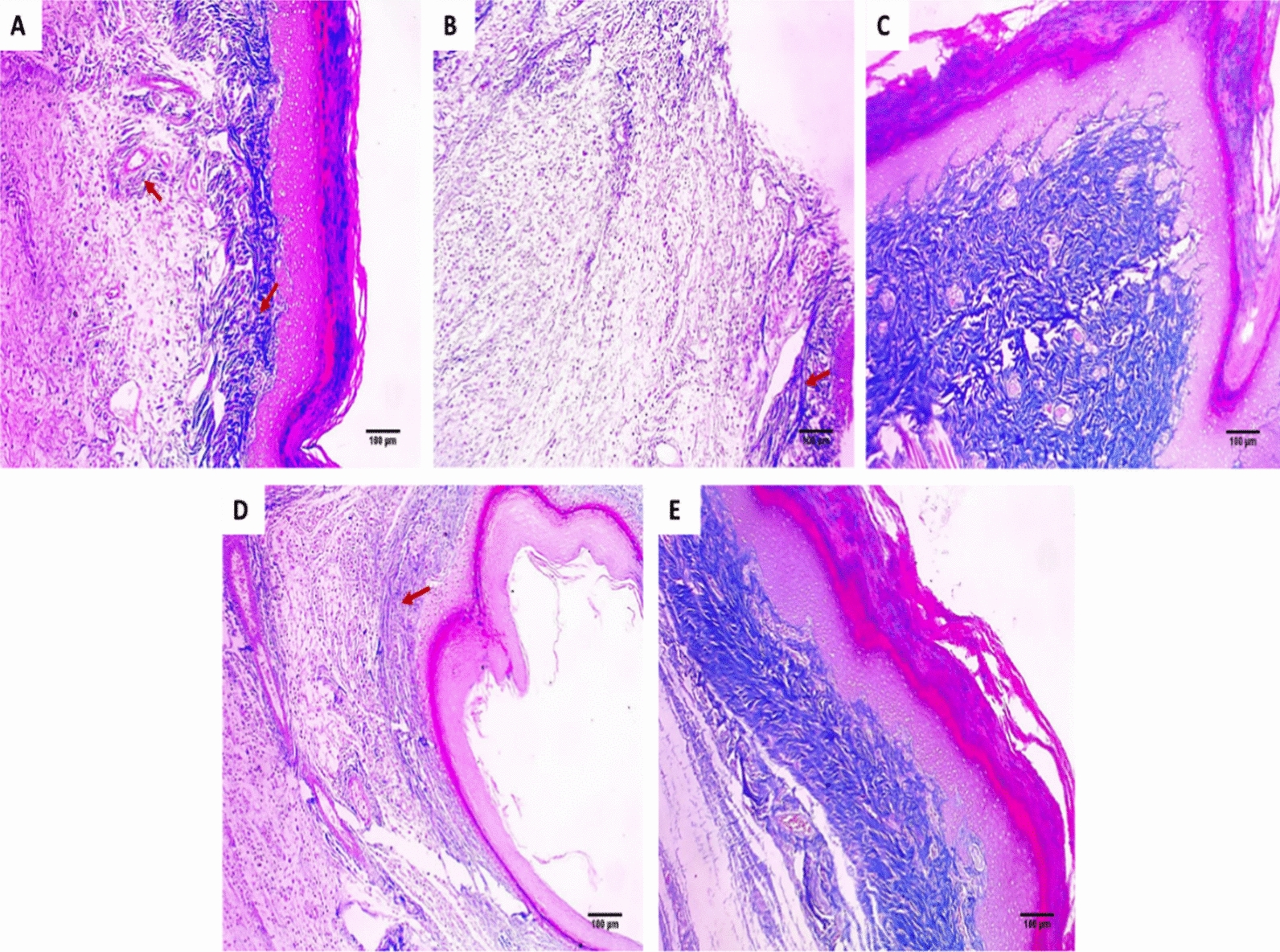
Paw sections stained with Masson’s trichrome stained: **A** normal control group revealing dermal bundles of thin blue stained collagen fibers (red arrows) (×100). **B** Positive control group revealing focal collagen bundles (red arrow) (×100). **C** Standard drug group revealing a marked increase of collagen thickness (×100). **D** Endophytic fungi group (100 mg/kg) revealing mild increase of collagen thickness (×100). **E** Endophytic fungi group (200 mg/kg) revealing a moderate increase of collagen thickness (×100)

The paw edema sections of the five experimental groups were stained using H&E and Masson's trichrome stain (Figs. 4 and 5).

#### Immunohistochemical assessment

**Fig. 6:**
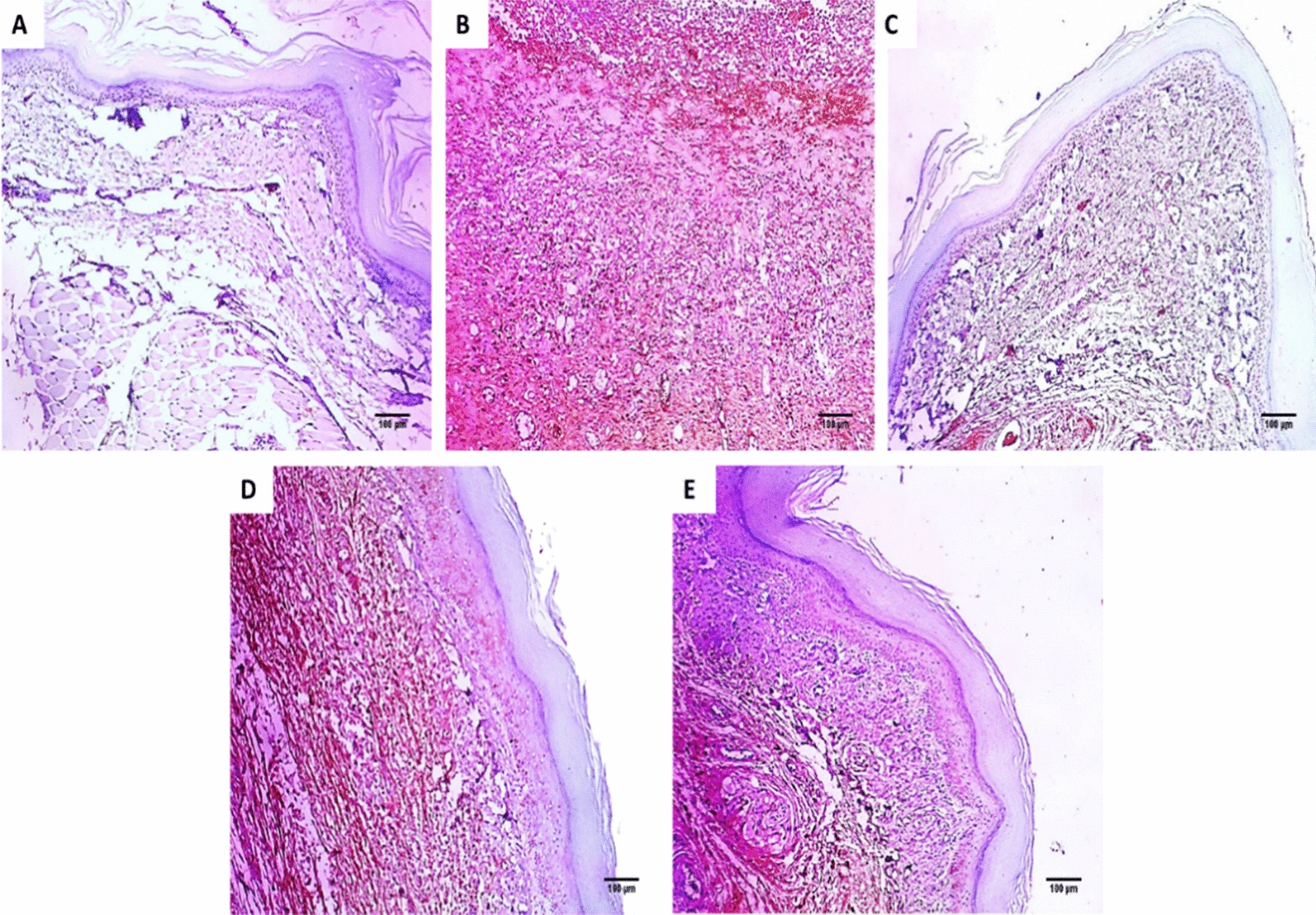
Paw sections immunostained with COX-2 monoclonal antibodies: **A** normal control group revealing negative immunostaining (0.18%) with score 0 (×100). **B** Positive control group revealed strong positive immunostaining (83.16%) with a score of 3 (×100). **C** Standard drug group revealing mild positive immunostaining (8.6%) with score 1 (×100). **D** Endophytic fungi group (100 mg/kg) revealing a strong positive immunostaining (74.22%) with a score of 3 (×100). **E** Endophytic fungi group (200 mg/kg) revealed moderate positive immunostaining (35.285%) with a score of 2 (×100)

**Fig. 7:**
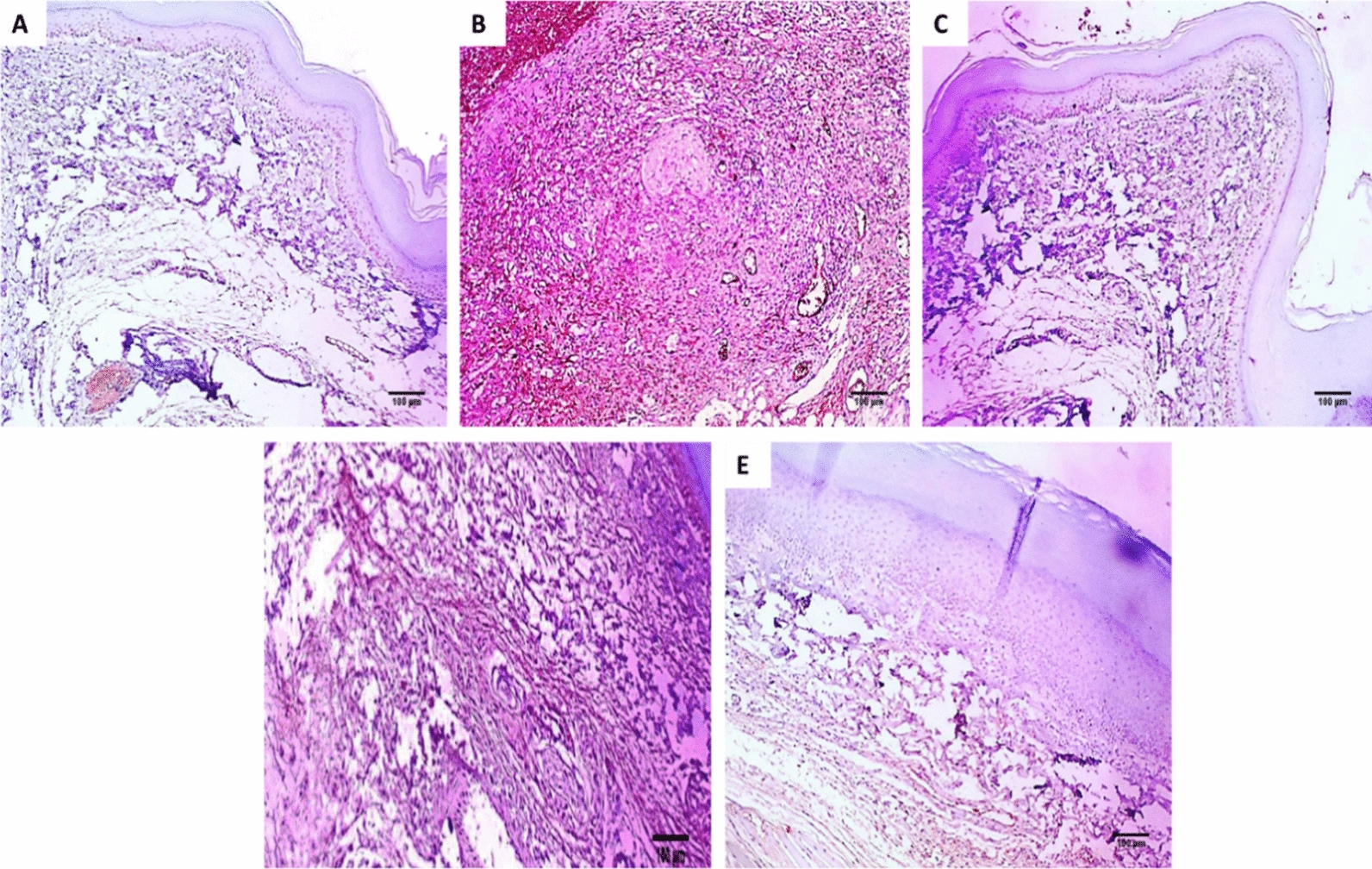
Paw sections immunostained with TNF-α monoclonal antibodies: **A** normal control group revealing negative immunostaining (0.26%) with score 0 (×100). **B** Positive control group revealed strong positive immunostaining (76.34%) with a score of 3 (×100). **C** Standard drug group revealing negative immunostaining (0.47%) with score 0 (×100). **D** Endophytic fungi group (100 mg/kg) revealing moderate positive immunostaining (26.18%) with a score of 2 (×100). **E** Endophytic fungi group (200 mg/kg) revealing mild positive immunostaining (5.62%) with a score of 1 (×100)

Immunostained COX-2 and TNF-α paw sections of the experimental groups are revealed in Figs. 6 and 7.

#### Biomarkers

**Fig. 8:**
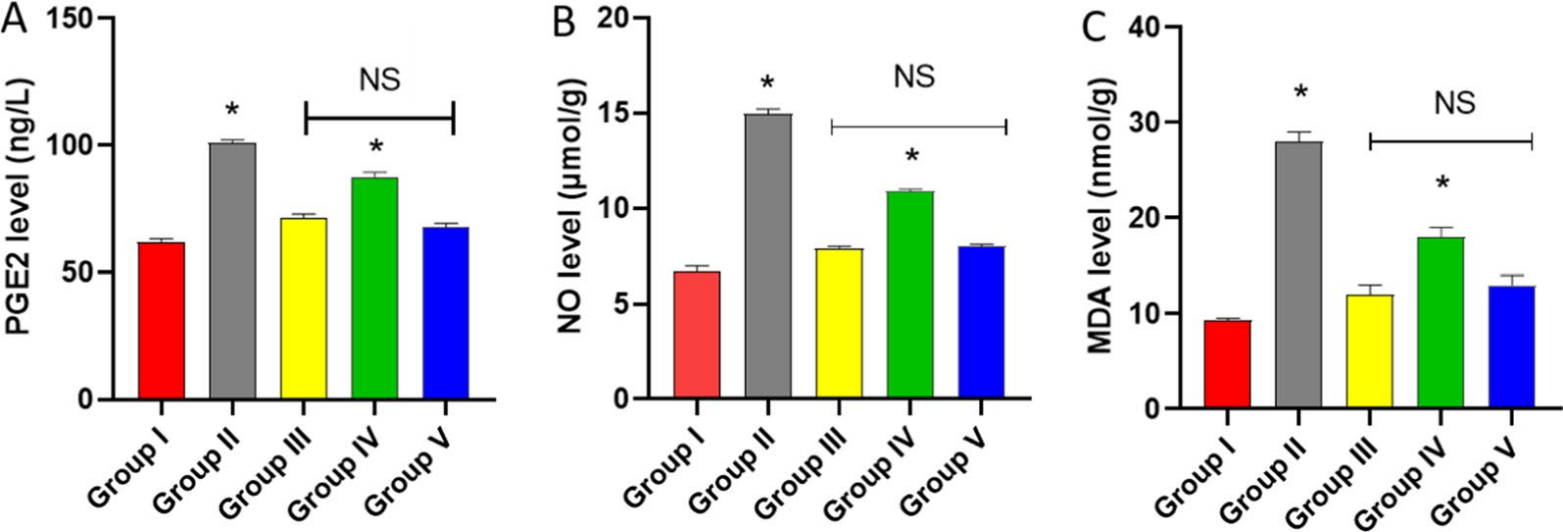
Influence of the endophytic fungi on the levels of **A** PGE2, **B** NO, and **C** MDA. The symbol (*) designates a substantial difference (*p* < 0.05) between group V and groups II and IV. NS designates a non-substantial difference (*p* > 0.05) between groups III and V

The influence of the endophytic fungi was studied on the level of PGE2 by ELISA as well as NO and MDA in using colorimetric kits in the paw tissues (Fig. 8).

#### qRT-PCR

**Fig. 9:**
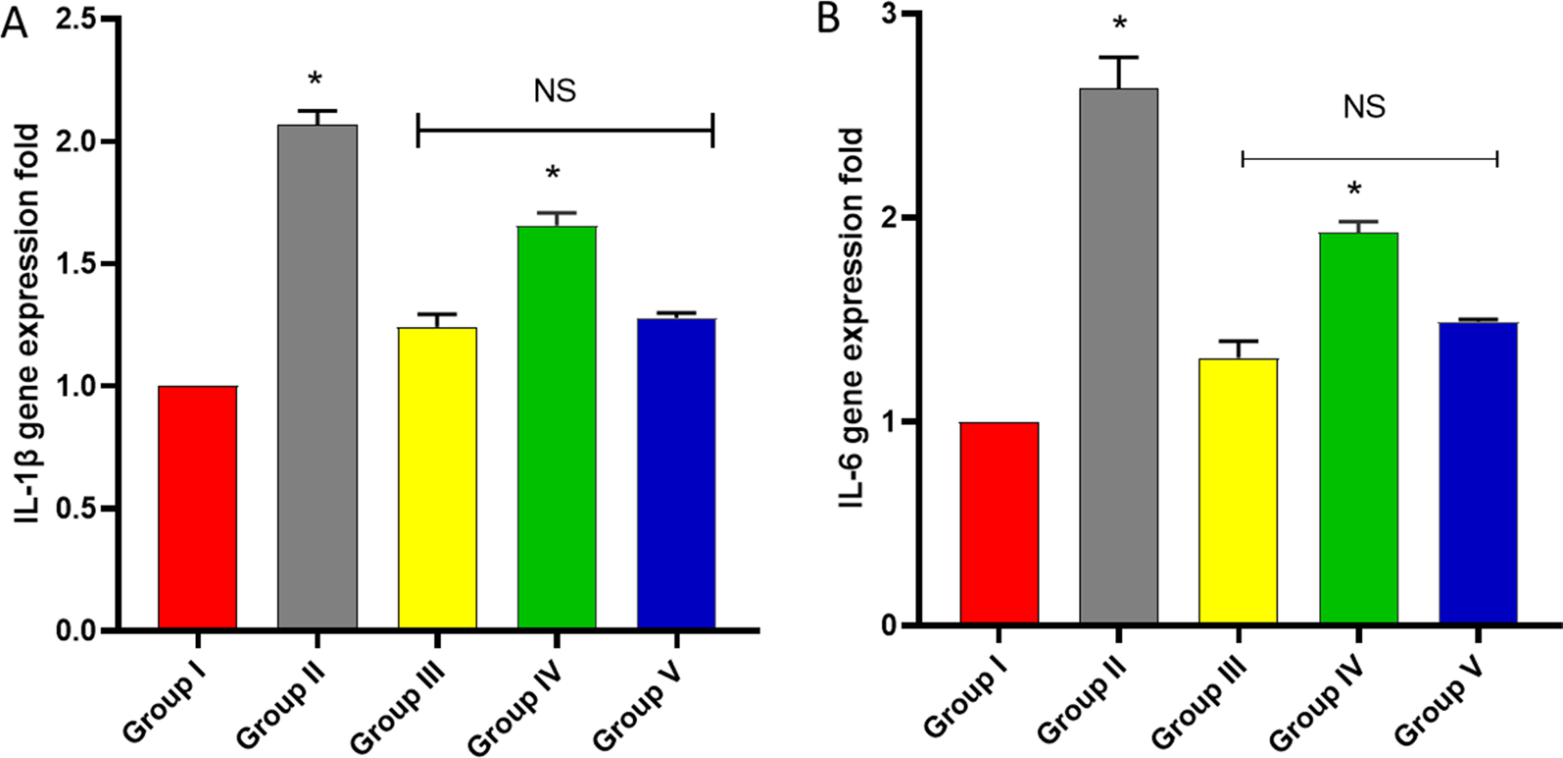
Influence of the endophytic fungi on **A** IL-1β and **B** IL-6 gene expression. The symbol (*) designates a substantial difference (*p* < 0.05) between group V and groups II and IV. NS designates a non-substantial difference (*p* > 0.05) between groups III and V

The influence of the endophytic fungi on the IL-1β and IL-6 expression levels in the paw tissues was elucidated by qRT-PCR (Fig. 9).

## Discussion

Endophytic microorganisms, particularly fungi, have a metabolic potential to generate various bioactive compounds [28]. Herein, LC–ESI–MS/MS studies of both positive and negative ionization modes of *P. brefeldianum* endophytic fungus revealed 27 bioactive metabolites compounds, 14 in the positive ionization mode and thirteen in the negative mode. The detected metabolites are of several phytochemical classes, including amino acids, carboxylic acids, and other derivatives, in agreement with the previous literature [29–31]. The major identified compounds in positive mode are agmatine, glycine-Betaine, choline, and carnitine. On the other hand, compounds; linoleic acid, malic acid, uridine, and citric acid were identified as majors in the negative mode. The anti-inflammatory potential of *P. brefeldianum* endophytic fungus was elucidated by the injected carrageenan into the paw of the studied rats. This model is commonly employed to study the anti-inflammatory potential of the plausible active compounds, as carrageenan can induce the discharge of many inflammatory and oxidative stress mediators involved in the inflammatory process [32, 33].

Edema is a crucial characteristic of inflammation that results from the accumulation of vast amounts of liquids in the tissues. It has a deleterious consequence on the function and metabolism of the tissues [34]. Throughout the inflammatory process, many inflammatory markers are produced which mediate the inflammatory process, like TNF-α, PGE2, IL-1β, and IL-6. This is in addition to oxidative stress markers like MDA and NO [35] produced by the inflammation cell-like macrophages [36]. Thus, to study the anti-inflammatory potential of certain compounds, we need to explore their effect on such mediators. Several bioactive agents formed by the endophytic fungi possess the ability to lessen the reactive oxygen species (ROS) levels [37]. Here, the endophytic fungus was found to have a remarkable effect (*p* < 0.05) on decreasing the levels of NO and MDA, which are considered important markers of oxidative stress [38]. A previous study revealed that the fungi obtained from *Bauhinia variegate* leaves exhibited antioxidant potential [39].

Many interleukins like IL-6 and IL-1β propagate the inflammatory process [40]. Thus, we studied the impact of the endophytic fungus on the gene expression of these mediators by qRT-PCR in the paw skin tissues. Remarkably, there was a considerable decline in the gene expression of these interleukins in the endophytic fungi treated group (200 mg/kg) in comparison with the positive control and the 100 mg/kg treated groups.

The COX-2 pathway involves the formation of PGE2, which is vital in the inflammatory process [41]. In the current study, the positive control exhibited a rise in the percentage of the positive COX-2 and TNF-α immune reactive cells. Such a finding was lessened via treatment with the endophytic fungi (200 mg/kg). Furthermore, the histological assessment of the paw sections stained with H&E and Masson's trichrome stains showed that the endophytic fungi (200 mg/kg) treated group had no inflammation compared with the positive control and the group treated with endophytic fungi (100 mg/kg). Previous studies revealed the anti-inflammatory potential of endophytic fungi like the mangrove endophytic fungus *Amorosia* sp. [42] and *Diaporthe* sp. [43].

## Conclusion

The current study’s findings displayed that *P. brefeldianum* endophytic fungus isolated from *A. hispida* leaves demonstrated an efficient anti-inflammatory action, at a concentration of 200 mg/kg, in the utilised carrageenan-induced paw edema model. The modified histological and immunohistochemical features assured this of the paw skin sections, in addition to the decline in the inflammatory and oxidative stress biomarkers revealed by ELISA and qRT-PCR.

The current investigation was designed to provide insights into the anti-inflammatory action of crude metabolites from endophytic fungi using carrageenan-induced inflammation in rats. Our study showed that *P. brefeldianum* crude extract exhibited a potent anti-inflammatory activity. These results suggest that the active endophyte identified from the present study can produce anti-inflammatory agents. This confirms that this endophytic fungus can be a reliable source for bioactive compounds with greater intrinsic chemo diversity. An important limitation of the current study is we didn’t isolate the bioactive compounds from the endophytic fugal extract. Further anti-inflammatory-guided fractionation is ongoing to purify and identify active compounds in a future study that may serve as promising starting point for developing and discovering new and potent pharmacological agents.

## Supplementary Information

**Additional file 1: Table S1.** The sequences of the utilized primers. **Figure S1.** Phylogenetic tree of *P. brefeldianum* endophytic fungusbased on 18S rRNA sequencing. **Figure S2.** The total ion chromatogramsof *P. brefeldianum* extract Negative ion mode. **Figure S3.** The total ion chromatogramsof *P. brefeldianum* extract Positive ion mode.

## Data Availability

The data presented in this study are available on request from the corresponding author. The data are not publicly available due to confidentiality policies.
