# Supplementary material for: Anti-inflammatory potential of Penicillium brefeldianum endophytic fungus supported with phytochemical profiling
Source: Microb Cell Fact. 2023 Apr 27;22:83. doi: 10.1186/s12934-023-02091-5 (PMC10141907; doi:10.1186/s12934-023-02091-5)
Supplement: Supplementary file 1 — Additional file 1: Table S1. The sequences of the utilized primers. Figure S1. Phylogenetic tree of P. brefeldianum endophytic fungusbased on 18S rRNA sequencing. Figure S2. The total ion chromatogramsof P. brefeldianum extract Negative ion mode. Figure S3. The total ion chromatogramsof P. brefeldianum extract Positive ion mode. [file 12934_2023_2091_MOESM1_ESM.docx]

**Table S1.** The sequences of the utilized primers.

| Gene | Sequence |
| --- | --- |
| Beta actin | Forward 5'-GTCAGGTCATCACTATCGGCAAT-3'  Reverse 5'- AGAGGTCTTTACGGATGTCAACGT-3' |
| IL-1β | Forward 5'-CACCTCTCAAGCAGAGCACAG-3'  Reverse 5'-GGGTTCCATGGTGAAGTCAAC-3' |
| IL-6 | Forward 5'-GCCCTTCAGGAACAGCTATGA-3'  Reverse 5'-TGTCAACAACATCAGTCCCAAGA-3' |


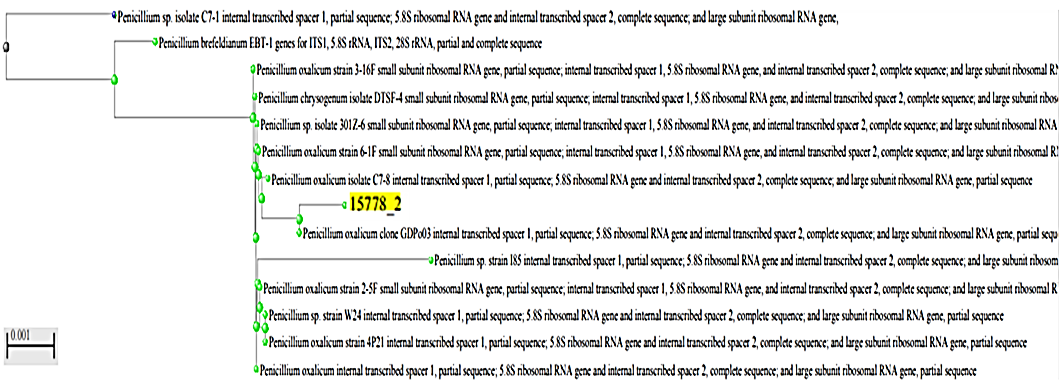


**Figure S1.** Phylogenetic tree of *P. brefeldianum* endophytic fungus (with yellow highlight) based on 18S rRNA sequencing.

**Figure S2.** The total ion chromatograms (TIC) of *P. brefeldianum* extract Negative ion mode.

**Figure S3.** The total ion chromatograms (TIC) of *P. brefeldianum* extract Positive ion mode.
